# Sports Video Classification Framework Using Enhanced Threshold Based Keyframe Selection Algorithm and Customized CNN on UCF101 and Sports1-M Dataset

**DOI:** 10.1155/2022/3218431

**Published:** 2022-12-08

**Authors:** M. Ramesh, K. Mahesh

**Affiliations:** Department of Computer Applications, Alagappa University, Karaikudi, Tamil Nadu, India

## Abstract

The computer vision community has taken a keen interest in recent developments in activity recognition and classification in sports videos. Advancements in sports have a broadened the technical interest of the computer vision community to perform various types of research. Images and videos are the most frequently used components in computer vision. There are numerous models and methods that can be used to classify videos. At the same time, there no specific framework or model for classifying and identifying sports videos. Hence, we proposed a framework based on deep learning to classify sports videos with their appropriate class label. The framework is to perform sports video classification using two different benchmark datasets, UCF101 and the Sports1-M dataset. The objective of the framework is to help sports players and trainers to identify specific sports from the large data source, then analyze and perform well in the future. This framework takes sports video as an input and produces the class label as an output. In between, the framework has numerous intermediary processes. Preprocessing is the first step in the proposed framework, which includes frame extraction and noise reduction. Keyframe selection is carried out by candidate frame extraction and an enhanced threshold-based frame difference algorithm, which is the second step. The final step of the sports video classification framework is feature extraction and classification using CNN. The proposed framework result is compared with pretrained neural networks such as AlexNet and GoogleNet, and then the results are also compared. Three different evaluation metrics are used to measure the accuracy and performance of the framework.

## 1. Introduction

The processing of images and videos is the core focus of computer vision research. A dynamic area of computer vision is video classification (VC) [1]. Due to its widespread use in automatic video analysis, video retrieval, and other similar kinds of applications, activity recognition is an essential topic [2]. The automatic classification of various sports using machine vision techniques is referred to as the classification of sports videos based on semantic information. Due to the huge demand for sports videos for training and development, the classification of sports training videos using machine learning and vision technologies has significant potential for commercial use [3]. However, video classification is still challenging work since it involves a huge number of data and processing steps [1]. After deep learning models became a booming technique for robotically identifying videos, this subject attracted greater attention [4]. The importance of accurate video classification is recognized by the huge amount of data available both online and in repositories. Convolutional neural networks (CNN) were investigated and proven to be useful tools for the categorization and analysis of image or picture material [2]. CNN has been widely used for object segmentation, detection, and so on. Similarly, video content analysis, video processing, and classification are also implemented with the convolutional neural network. CNN has been used with the functions and techniques of deep learning, which have also achieved good results in computer vision applications.

In recent years, a video categorization task has shown tremendous success. This study gives a very detailed and technical strategy for sports video classification in order to acknowledge the significance of the video classification task and to highlight the accomplishments of deep learning models for this work. Generally, people all over the world generate and handle huge amounts of video and share it through social media like Facebook, WhatsApp, Signal, and so on. Currently, on YouTube alone, over one billion hours of video are being watched by different people every single day [4]. Businesses like Google AI are investing in several challenges to find creative solutions to difficult issues with limited resources. Google AI has released a public dataset called YouTube-8M, featuring millions of video attributes and over 3700 labels, to promote the development of autonomous video categorization tasks. All of these initiatives highlight the requirement for an effective video categorization model [4].

The ultimate aim of this work is to classify sports videos based on their content using the proposed model. This model begins with preprocessing the input sports video. Preprocessing includes both frame extraction and noise reduction. Then, the keyframes are extracted using the proposed enhanced key-frame extraction algorithm. Finally, keyframes are given to CNN to extract feature sets based on its trained knowledge of CNN. Given the input, sports video is categorized as a specific class at last.

This work's primary contribution is as follows:

- Frame extraction and noise reduction are carried out in preprocessing.
- keyframe selection technique is used to extract key-frames.
- The framework classifying sports videos are built using convolutional neural networks and deep learning techniques.
- Run the framework using the test data.

The efficiency of the suggested technique is then confirmed by comparing the findings to an existing model. The suggested framework for classifying sports video is novel in that it uses a specially designed keyframe selection method, a fuzzy adaptive window-based mean filter (FAWMF) to eliminate noise, and hyper-parameters that are adjusted depending on the two datasets stated above. There are many frameworks or models available for categorizing videos in general. As an illustration, consider the classification of moving objects, the recognition of human movement, action recognition, etc. They are mentioned in the literature review section in detail. Recent advancements in deep learning models have shown how effective these methods can be at categorizing videos. However, the majority of the popular deep learning models for video classification have been mostly adapted from those in the image/speech domains. Existing works are stated with the name of the model, optimization techniques or algorithms, a dataset, and the outcome of the model. The framework proposed in this research is specifically for sports video classification and also delivers outstanding performance and good accuracy, and the results are compared with the existing work.

The rest of this paper is organized as follows: video classifications and literature reviews are included in Section 2. The deep learning architecture is given in Section 3, including CNN and its layers. Datasets and their characteristics were discussed in Section 4.  Section 5 offers a proposed method with its framework architecture; the experimental findings and comparisons to related works are presented in Section 6; the conclusion and future works are presented in Section 7.

## 2. Literature Review

Atiqur Rehman and Samir Brahim Belhaouari explored a review of video classification in different categories of approaches, like hand-crafted approach and 2D-CNNs. 3D-CNNs, spatiotemporal convolutional networks, and recurrent spatial networks [4]. Moumita Sen Sarma et al. used to categorie traditional sports videos from Bangladesh by removing both the spatial and temporal characteristics from the recordings [5]. The development of a scratch model using the two most popular deep learning techniques, convolutional neural network (CNN) and long short-term memory (LSTM)is a fundamental contribution of this paper [5]. Malik Tabish et al. worked to invent a convolutional neural network (CNN)-based model for sports activity recognition with similar content. The pretrained VGG16, VGG19, ResNet50, and Inception V3 models are used to train the model, and the clustered cricket video frames from the specifically produced dataset are used to test it [2]. Na Feng et al.'s Soccer Dataset for Shot, Event, and Tracking (SSET) was created to create a soccer dataset that may be used for player tracking, shot segmentation, and soccer event detection research [6].

Yunjun Xu et al. presented the event matching method is used to match the convolutional neural network output to complete the sports training video classification [3]. Hana et al. present an efficient keyframe extraction method. By applying the modularity concept to graph clustering, the keyframe selection is carried out. The results of the tests demonstrated that the suggested method is effective in extracting keyframes that maintain the pertinent video content without duplication [7]. Shahil et al. propose a sports identification system using a more complex CNN model that includes fine-tuning and a fully linked layer. Five different sports groups are categorized using photographs and videos. In this study, we employ an image-based video categorization approach [8]. The notable papers are mentioned in Table 1. Based on the related research, this framework takes sports video classification as the core concept.

## 3. Deep Learning Architectures

Machine learning is a subset of artificial intelligence, while deep learning is a subset of machine learning. It is a very important element of data science and certainly includes statistical approaches and predictive models. Data scientists are mostly preferred for deep learning architecture for the task of collecting the data, analyzing the data, and also interpreting a very large amount of data. The biggest advantage of using deep learning is that it makes the above process smarter, faster, and easier. DL is a class of algorithms and topologies, not a single method, which can be used to solve a variety of problems. Many architectures and algorithms are used in deep learning. Generally, deep learning architecture is classified into two categories. There is supervised and unsupervised learning. LSTM and CNN fall under supervised learning. These two are the oldest approaches and most widely used architectures in various applications. In this paper, we experimented with the pretrained networks AlexNet and GoogleNet, and the results are compared with the proposed model.

### 3.1. CNN—Convolutional Neural Network

CNN is a class of ANN in deep learning. It is especially useful for analyzing visual content like images and videos. AlexNet, DesnseNet, GoogleNet, LeNet, ResNet, and VGGNet are predefined and widely used architectures for image and video content analysis [4]. The CNN model covers one or more layers of subsampling and convolution, which go behind the fully connected layers, which can be single or multiple, and an output layer [2]. CNN has been attested to be the most efficient one when it comes to classification problems [16]. It is a great model for both image and video analysis. Since CNN has become more popular in the past few years, and this is the very basis for modern computer vision-based applications like video embedding, encryption, classification, and so on.

CNN can be partitioned into different types of layers, and each layer performs various missions. Among these layers, one of the most important is the convolutional layer. It handles feature extraction with the support of convolution maps. Then, the remaining layers are the input layer, the ReLU layer, the pooling layer, and the fully connected layer.

#### 3.1.1. Input Layer

The working principle of the input layer in CNN is similar to the way we use it to give input for a model. The only difference is that it takes three-dimensional values. The height and width of the layer represent the horizontal and vertical pixels of the image, respectively, while the depth represents the RGB color channel values.

#### 3.1.2. Convolution Layer

The major component of CNN is the convolution layer. This layer is where the convolution occurs, which means the layer tries to find features in the input like images or frames in the case of videos. Frames and images are constant, which means that one component of the image is created similarly to all other components [2]. As a result, the training function in one region can be replicated in another region. The most important features are going to be found for classification with the help of filters. Filters are passed over the image or frame. The result of this process is known as feature extraction. The following variables are used to extract features:

- Activation map size
- Filter size
- Stride
- Padding

**Figure 1:**
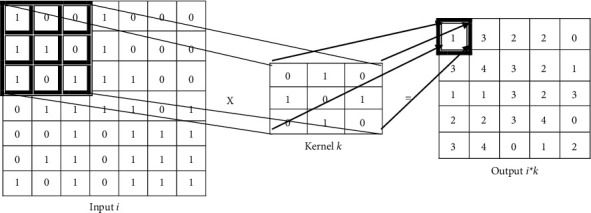
Activity of convolutional layers.

More convolution layers in a CNN are also possible. When we need high-level features, we need to use more than one convolution layer. In the first layer, the network could detect simple edges, and then in the next layer, those edges could be filtered into simple shapes, and so on. Formal convolution layer activity is shown in Figure1.

#### 3.1.3. The ReLU Layer

In general, the ReLU layer is useful for activation functions. It helps the network maintain the minimum computational cost.

#### 3.1.4. Pooling Layer

**Figure 2:**
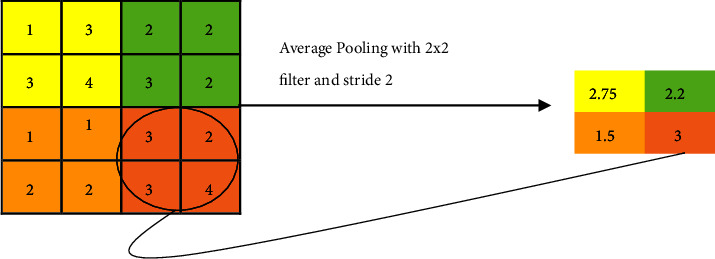
Average pooling.

**Figure 3:**
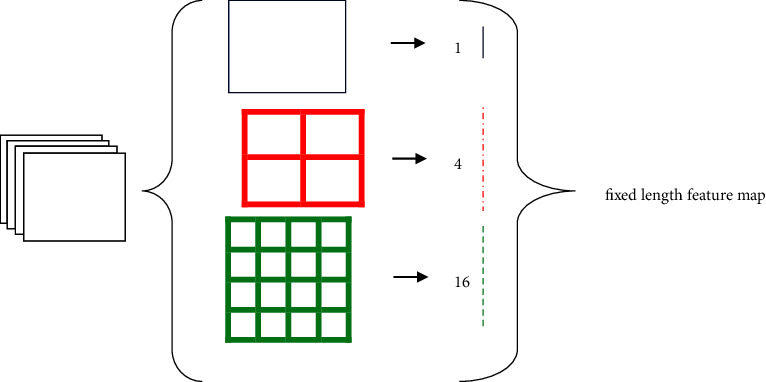
Spatial pyramid pooling [17].

Subsampling or downsampling is nothing but a pooling layer. The pooling layer is a mediator between two consecutive convolution layers. The method of downsampling of an image is well-known as pooling. The convolution layer (CL) output is subsampled for a single output using a small amount of it as an input [2]. Popular pooling methods are average pooling, max-pooling, mixed pooling, Lp pooling, stochastic pooling, spatial pyramid pooling, and region of interest pooling. In general, pooling reduces the number of parameters to be calculated but makes the network constant or equal in form, size, and scale translations [2]. An average or mean pooling layer achieves downsampling by separating the input into rectangular pooling regions as well as computing the average values of each region. The average pooling example is shown in Figure 2. SPP (Spatial Pyramid Pooling) removes the fixed size constraint of the network, which pools the features and generates fixed-length outputs that are then fed into the fully connected layers. The working principle of spatial pyramid pooling is shown in Figure 3. Figure 3 may be analyzed using SPP, which has a number of pooling layer scales that can be applied to convolutional layer features of any size and ultimately produce eigenvectors with fixed dimensions [18].

#### 3.1.5. Fully-Connected Layer

This layer is the only layer that is fully connected to the previous layer, and it is the last layer in CNN. It classifies the feature data extracted and downsampled in previous layers. It takes feedback from the previous layer and produces output.

## 4. Dataset

UCF101 is a dataset of realistic action videos with 101 action categories that were gathered from YouTube. The UCF50 dataset has been expanded to create this data collection. There are 50 activities in UCF50.13320 videos from 101 activity categories are included in the UCF101 data collection. With huge differences in camera movements, object appearance and position, object scale, viewpoint, cluttered backgrounds, illumination conditions, and other factors, UCF101 provides the most diverse range of sports in terms of actions. UCF101's major purpose is to promote more action recognition research by learning and exploring new realistic action categories.

The Sports-1M dataset contains over a million YouTube videos. The collection contains over a million videos, divided into 487 sports-related categorise with 1,000 to 3,000 videos each. By examining the text metadata connected with the videos, the YouTube Topics API is used to automatically categories the videos into 487 sports classes.

## 5. Methodology

**Figure 4:**
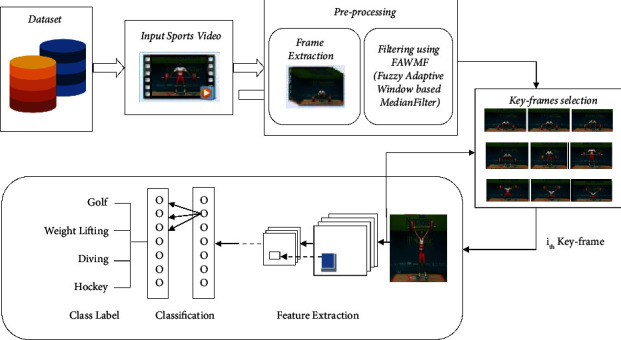
Proposed framework for sports video classification.

A video is a three-dimensional signal in which the horizontal axis corresponds to the frame width and the vertical axis corresponds to the frame height; the third axis depicts the evolution of frame content over time.  Figure 4 demonstrates the framework for the proposed sports video classification. Data collection and preprocessing proceeded before the keyframe extraction task. Then, the dataset is divided into train and test for CNN in the ratio of 80 : 20. The suggested model accepts sports videos as an input and generates class labels as an output. Two benchmark datasets, UCF101 and Sports1-M, are used to train and test the suggested framework.

### 5.1. Preprocessing

Preprocessing is the very first step for every research and its implementations. Hence, sports video classification also begins with preprocessing. It involves a process known as frame extraction, which entails converting the given sports video into frames. As mentioned in section, UCF101 has 101 different categories of sports videos with sports names as folder names.

(1) $$\begin{array}{c} SV_{1}=\left\{F_{1},F_{2},F_{3}\ldots \ldots \;F_{N}\right\}\;where\;N=101\text{.} \end{array}$$

In (8), *SV*_1_ is sports video category1, and *F*_1_, *F*_2_, *F*_3_,…, *F*_101_ are various folders for different sports categories. Each and every folder has many types of videos with different styles.

(2) $$\begin{array}{c} F_{i}=\left\{V_{1},V_{2},V_{3\;},\ldots .V_{n}\right\}where\;i=1\;to\;n\text{.} \end{array}$$

#### 5.1.1. Frame Extraction

In this model, frame extraction is one of the major courses of action in video preprocessing. A video is a collection of pictures that are taken and then shown repeatedly. However, a single video frame, or image, is obtained by pausing the sequence at a particular frame. The mathematical approach for frame extraction is mentioned in equation (3). Frame conversion is the very first course of action in the sports video classification model. This model acquires sports video as an input, then the same is converted into frames. Once the input sports video is converted into frames, the converted frames are passed to the next stage, which is known as keyframe selection or extraction.  Table 2 shows the properties and their respective values of an input sports video. The total frames from the original video file are extracted and stored in a specific location. From the following equation (3), *V*_*i*_ represents each video from the dataset. *f*_*i*_ is the number of frames in a video, which are indexed from 1 to *n*. *n* is nothing but the number of frames. There are a large number of sports video datasets available on social media and other sources. But very few datasets are considered standard. In Table 3 some sample benchmark datasets for sports video are listed.

(3) $$\begin{array}{c} F\left(i\right)=V_{i}\left(f_{i},f_{i+1},f_{i+2},f_{i+3},\ldots \ldots ,f_{n-3},f_{n-2},f_{n-1},f_{n}\right)\text{.} \end{array}$$

#### 5.1.2. Noise Reduction Using FAWMF

When video signals are acquired, transmitted, and received, noise is a significant element that can significantly reduce the quality of the signals [19]. We may not get an exact outcome because of a noisy image or frames in an application. Noise reduction is a highly attractive process for better video quality. Since interframe noise reduction is efficient for areas of video frames where there is no motion, we cannot use inter-frame noise reduction in the proposed framework. We can apply a spatial-temporal filter, which is successful in removing noise [20]. In general, such filters have the capability to decrease noise efficiently. But uncertainly, it may cause blurring effects on the input frames. The fuzzy adaptive median filtering (FAMF) technique is useful for the preprocessing stage, which removes the noise in the video frames [21]. In order to do spatial processing and identify the pixels that are impacted by impulse noise, FAMF is primarily used [21].

In this paper, a fuzzy adaptive window-based Mean Filter (FAWMF) is used for preprocessing the sports video after the frames are extracted. The pixels are categorized as “noise” based on how each pixel in the picture is compared to its neighbors. After the noise intelligence test, these pixels are then replaced by the value of mean pixel value in accordance with their neighbors. FAWMF improves the quality of video frames and removes impulse noise.

(4) $$\begin{array}{c} f^{\prime \left(x,y\right)}=\overset{i=1}{\underset{i=-1}{\sum }}.\overset{j=1}{\underset{j=-1}{\sum }}f\left(sum\left(x,i\right),sum\left(y,j\right)\right)*w\left(i,j\right). \end{array}$$

Algorithm 1 fuzzy adaptive window-based mean filtering follows these steps to apply the filter to each frame in a sports video. Initialize the window size *w*(*i*, *j*), where *i* and *j* = 5. Then, travel the filter matrix over the video frame *f* and *w*(0,0) must go along with the current frame position (*x*, *y*). Apply the product term to each frame element using the corresponding filter coefficient *w*(*x*, *y*). Find average of sum of products. Finally, the current position value is replaced by an average value.

### 5.2. Keyframe Selection

**Figure 5:**
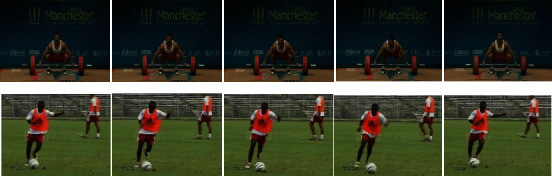
Subsequent frames with similar content.

Keyframe selection is one of the most crucial and significant works in the video classification model since the processing of all the frames in a video will boost both time and space complexity, which may degrade the performance of the model.  Figure 5 shows the subsequent frames with similar content. The content of the frames and their features are similar, so we do not want to train and test all the similar frames. A 00.05 minutes spots video may have a minimum of 50 to 55 frames. This model was trained and tested with the UCF101 and Sports-1M datasets. The two datasets have more than 101 different sports categories separately. The foremost intention of keyframe extraction is that there be no significant variation between consecutive frames. The proposed approach for keyframe extraction is composed of two steps: first, identifying the candidate frame using the skip factor (SF) [1], which is stated in Algorithm 1. Because all the frames in a video do not to be processed, consecutive frames may have common objects and features. Then, apply an enhanced threshold-based frame difference (ETFD) algorithm, which is mentioned in Algorithm 2, for identifying keyframes. The following algorithm extracts 24% of frames from a video. As a result, processing time will be reduced while model performance will improve.

A mathematical formula for candidate frame selection is given as follows:

(5) $$\begin{array}{c} V=\left\{f_{1},f_{2},f_{3},\ldots f_{n}\right\}\text{,}\\ CFs=\left\{cf_{1},cf_{2},cf_{3},\ldots cf_{n}\right\}\text{.} \end{array}$$

Once candidate frames are extracted, an apply enhanced threshold-based frame difference algorithm for keyframe extraction. The Algorithm 3 takes candidate frames as input and applies the frame difference method to extract keyframes. A mathematical formula for keyframe selection is given as follows:

(6) $$\begin{array}{c} FD\left({cf}_{i},{cf}_{i-1}\right)=\frac{1}{\;\mathrm{mod}\left(F\right)}\overset{n}{\underset{i}{\sum }}\mathrm{mod}\left(cfi\left(x,y\right)-cfi-1\left(x,y\right)\right),\\ FD\geq T\text{.} \end{array}$$

### 5.3. CNN

**Figure 6:**
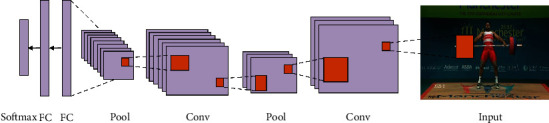
Typical CNN layers with an input frame [22].

**Figure 7:**
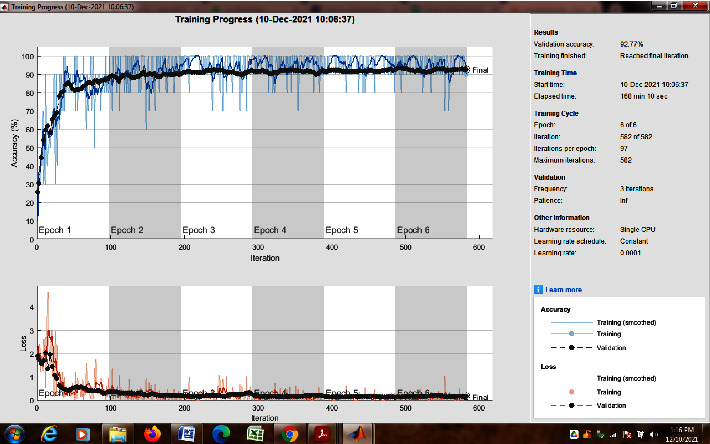
Training progress of the proposed model.

The convolutional layer is the most important element of the convolutional neural network (CNN). We applied CNN to classify objects in all the frames from the sports video dataset into various classes. We trained over 1,800 videos in the UCF101 dataset, which are identified with 91% accuracy. The working model of CNN is shown in Figure 6. CNN is always compiled with multiple layers, one after another. The convolutional neural network begins with convolution and pooling layers, which are mainly used for breaking down the input frames into features and studying them autonomously. The collected features are carefully transferred to the appropriate classes in the classification process [23]. The outcome of this development is fed to the next layer, known as the fully connected layer, which takes the final classification.  Figure 7 displays the training progress of the proposed model.

**Figure 8:**
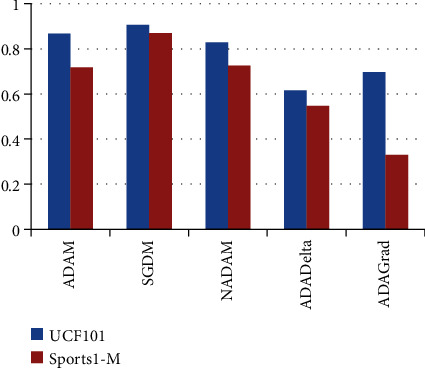
Comparison of various optimizers and their accuracy applied to the UCF101 and Sports1-M datasets.

For the above purpose, we apply ReLU, which initializes all of the negative values in a frame that is a two-dimensional matrix with zero. The value “zero” means the specific picture element has no value. The maximum value from the matrix is obtained using the max-pooling layer. The softmax algorithm is then used to assign decimal probabilities to all classes using the output of the fully linked layer.  Figure 8, illustrates the comparison of various optimizers and their accuracy when applied to the UCF101 and Sports1-M datasets.

## 6. Experimental Results

**Figure 9:**
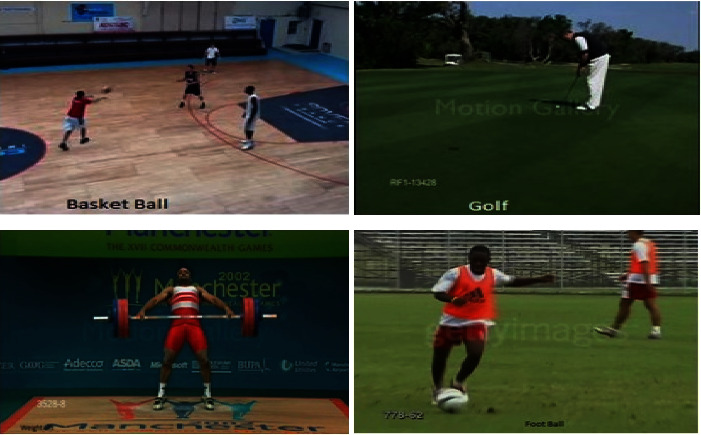
Accurate classification of random videos.

**Figure 10:**
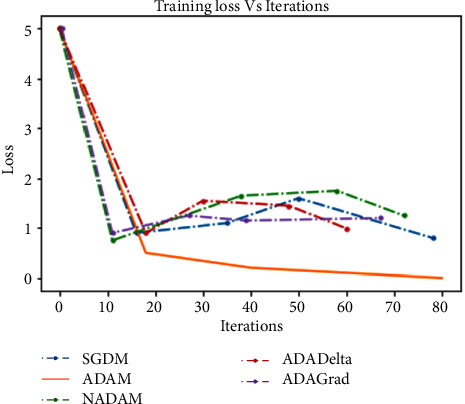
Training loss on a pretrained model using various optimizer with the UCF101 dataset.

**Figure 11:**
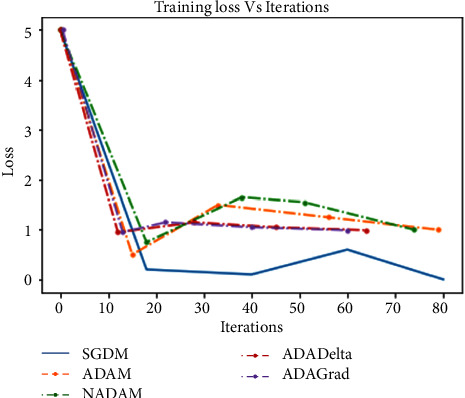
Training loss on a pretrained model using various optimizers with the Sports1-M dataset.

**Figure 12:**
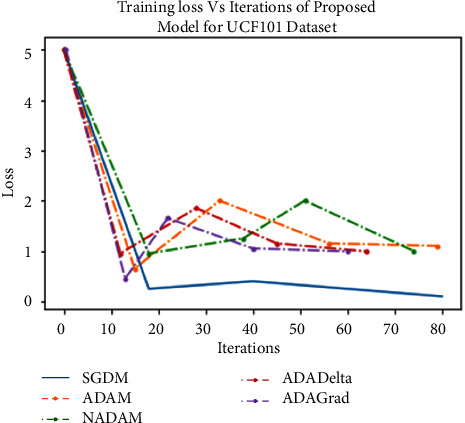
Training loss on the proposed model for the UCF101 dataset.

**Figure 13:**
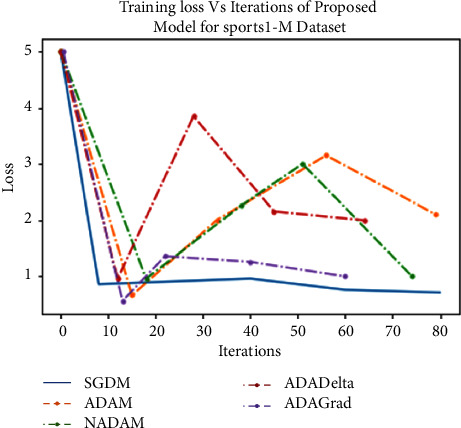
Training loss on the proposed model for Sports1-M dataset.

Experiments were performed on the UCF101 and sports1M datasets for sports video classification.  Table 4 shows the experimental setup, hyper-parameters, and their respective values used for the implementation. The reason for choosing the abovementioned dataset is that almost all kinds of sports are covered. UCF101 and sports1M datasets have 13,320 videos with 101 different classes and 11,33,158 videos with 487 classes, respectively. Initially, we evaluate the performance of the pretrained models like AlexNet and GoogleNet on the UCF101 dataset. Then, we fine-tune the abovementioned model with the Sports1-M dataset. Four sports videos are randomly intercepted from the UCF101 dataset and appropriately classified by the proposed model, and the result is shown in Figure 9. Figures 10 and 11 demonstrate training loss on a pretrained model using various optimizers with the UCF101 and Sports1-M dataset, respectively. Figures 12 and 13 indicate training loss on the proposed model for the UCF101 and Sports1-M datasets.

### 6.1. Evaluation Metrics

Space complexity, time complexity, precision, recall, f-measure, and compression ratio are various general metrics to measure the performance of an algorithm. Accuracy, *F*1-Score, precision, and recall are frequently used evaluation indices based on multilabel classification [25]. Since we also used only four metrics among the six metrics to measure the performance of the proposed model, the following mathematical method is used to calculate the compression ratio based on the keyframe selection. The compression ratio is determined by the uncompressed frames and compressed frames of a video.

(7) $$\begin{array}{c} Compression\;Ratio=\frac{N_{f}}{K_{f}}\text{,} \end{array}$$

where *N*_*f*_ is the total number of frames in a video and *K*_*f*_ is the number of keyframes selected to proceed. Recall and precision are employed in the fields of image classification, information retrieval, video classification, and segmentation.

(8) $$\begin{array}{c} Precision=\frac{NVCA}{TNVC}\text{,}\\ Recall=\frac{NVCA}{TNVDS}\text{,} \end{array}$$

where NVCA is the number of videos classified accurately, TNVC is the total number of videos classified, and TNVDS is the total number of videos in a dataset. A benchmark metric known as *F*-measure uses the harmonic mean to combine the precision and recall values into a single value.

(9) $$\begin{array}{c} F_{Measure}=\frac{2\;*\;Precision\;*\;Recall}{Precision+Recall}\text{.} \end{array}$$

These measurements are determined by categorizing shots correctly or incorrectly for each category [12]. Using the following equation, accuracy is calculated as the ratio of true positive and true negative samples to the total number of samples [2]:

(10) $$\begin{array}{c} Accuracy=\frac{TNVCA}{TNV}\text{,} \end{array}$$

where TNV is total number of videos and TNVCA total number of videos is classified accurately.

### 6.2. Results

The proposed framework's evaluation and performance are assessed using the evaluation metrics. Utilizing the UCF101 and sport1-M benchmark datasets, the framework is trained and tested.  Table5 includes information on the accuracy and various optimizers that are used to investigate performance. The UCF101 dataset with the SGDM optimizer produced great accuracy in terms of training and test performance, per the analysis of the abovementioned datasets. Comparatively speaking, the keyframe selection method and fuzzy adaptive window-based mean filter combination performs better. The framework offers improved performance with a less computational expense. As long as there is sufficient training data, feature extraction architectures using CNN (convolutional neural network) can outperform those using hand-crafted features.

### 6.3. Performance Comparison with Other Works

As a result, the majority of current architectures are still unable to handle the more complex nature of video data, which contains a wealth of information in the form of spatial, temporal, and audio cues [4]. Tables 6 and 7 show the results of the proposed model with a few pretrained network models. According to the investigation and the experiment results, the proposed framework produced better accuracy compared with the existing architecture with various optimizers. The results of the proposed framework are mentioned in Table 8. Finally, the training and test accuracy of the proposed framework is 92.77% and 93.59%, respectively for the UCF101 dataset. On the other hand, the training and test accuracy of the proposed framework is 82.52% and 89.75%, respectively, for the Sports1-M dataset. Also, the investigation results showed that the proposed framework obtained the best precision, recall, and f-measure at 96%, 94%, and 94%, respectively. Based on the results of training and testing, the suggested framework performs effectively in terms of both time and cost.

## 7. Conclusion and Future Work

Using the UCF101 and Sports1-M datasets, we proposed a framework for classifying sports videos in this research. The framework uses a sports video as its input and uses a number of intermediary processes to obtain the appropriate class label. The framework begins with frame extraction, keyframe selection using the skipping factor, and noise reduction, which are intermediate steps that are followed by the custom CNN. A personalized CNN was tested and trained using various optimizers, including SGDM, ADAM, NADAM, ADADelta, and ADAGrad. CNN is typically employed to extract the features and categories of the data in accordance with the objective of the research. The output of the suggested framework is then compared with the output of pretrained neural networks like AlexNet and GoogleNet, and the results are stated. The effectiveness and performance of the framework are evaluated using three separate indicators. Only the two benchmark datasets are used for training and testing the proposed system. Therefore, using the stated experimental setup, this can only give results that are adequate for these two benchmark datasets. This can be a drawback to the suggested framework. In the future, we may utilize effective keyframe extraction algorithms, different optimizers, and/or improved noise removal techniques to obtain better classification results for the same sports video classification problem [28] [29] [30].

## Figures and Tables

**Algorithm 1:**
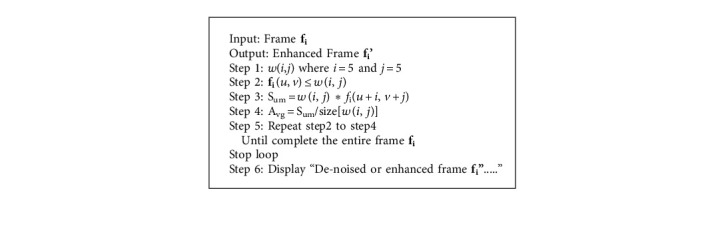
Fuzzy adaptive window based mean filter (FAWMF).

**Algorithm 2:**
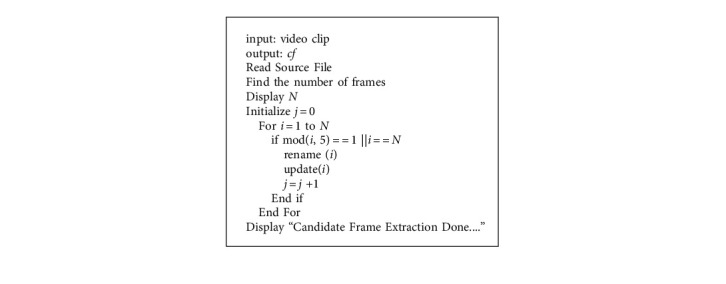
Candidate frame extraction using skip factor.

**Algorithm 3:**
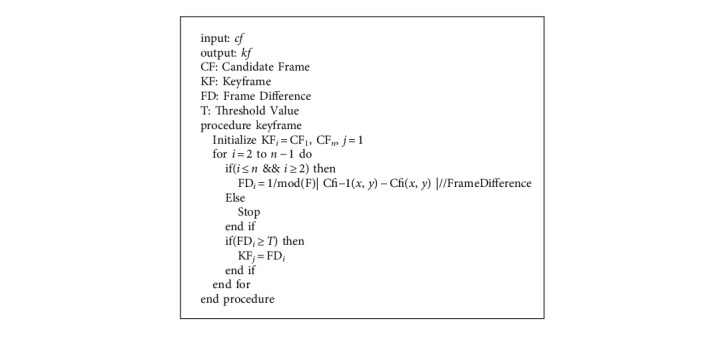
Enhanced threshold-based frame difference for keyframe selection.

**Table 1:** Summary of the related work.

Ref.	Year	Title	Model	Optimization technique/algorithm	Dataset	Class and accuracy	Outcome
[9]	2021	Olympic Games event recognition via transfer learning with photobombing guided data augmentation	AlexNet, VGG-16, ResNet-50	Transfer learning	OGED - Olympic games event image dataset	Multiclass and 90%	Olympic Game event recognition
[10]	2021	Categorization of actions in soccer videos using a combination of transfer learning and gated recurrent units	CNN, RNN, and soccer actions categorization	—	SoccerAct10	94%	10 soccer actions corner, foul, free-kick, goal-kick, long-pass, penalty, and so on.
[8]	2021	Sports recognition using convolutional neural networks with optimization techniques from images and live streams	Extended Resnet50 and VGG16	RMSProp, ADAM & SGD	5sports	Resnet50-83% and VGG16-95%	Sports event recognition.
[5]	2021	Traditional Bangladeshi sports video classification using deep learning method	CNN and LSTM	—	Traditional Bangladeshi sports video (TBSV), UCF sports, UCF101	5 classes and 99%	Bangladeshi sports Vido classification.
[3]	2021	A sports training video classification model based on deep learning	AlexNet		Various dataset	9 classes and 99%	Sports training video classification.
[4]	2021	Deep learning for video classification: A review	2D-CNNs, 3D-CNNs, handcrafted approaches.	—	—	—	Video classification in general
[2]	2020	Activity recognition framework in sports videos	Deep learning	K-means clustering	YouTube, cric-info	Multiclass	Frames extracted
[6]	2020	SSET: a Dataset for shot segmentation, event detection, player tracking in soccer videos	DevNet, VGG LSTM replay, LRCN, GoogLeNet	—	Soccer Dataset for Shot, Event, and Tracking (SSET)	Multiclass	Shot segmentation, event detection, player tracking
[1]	2020	Video event classification based on two-stage neural network	CNN and RNN	Transfer learning	UCF101, HMDB51 and CCV	Multiclass	Video event classification
[11]	2020	A K-means clustering approach for extraction of keyframes in fast-moving videos	—	Shot boundary detection, keyframe extraction		Three classes 23.52%, 14.11%, and 6.62%	Keyframe extraction
[12]	2019	Shot classification of field sports videos using an AlexNet convolutional neural network	AlexNet with the proposed framework	—	ESPN, star sport, sky sports, ten sports, etc.	Multiclass 94.07%	To classify the shots into long, medium, close-up, and out-of-the-field shots.
[13]	2019	Video genre identification using clustering-based shot detection algorithm	SVM, CNN	K-mean, K-medoid		Two classes and 90%	An audio talk show or another video
[14]	2019	Keyframe extraction based on HSV histogram and adaptive clustering	—	K-means, density peak clustering algorithm (DPCA), partition based clustering, I-frame	—	—	Keyframes
[7]	2018	Keyframe extraction for video summarization using local description and repeatability graph clustering	—	Graph clustering	Open video project (OVP) and YUV video sequences	—	Video summarization
[15]	2015	Real-time event classification in field sport videos	—	Decision tree, feed-forward neural network	Ground truth dataset together with an annotation technique	—	Event identification

**Table 2:** Dataset UCF101's properties and values.

Properties	Values
Length	00.05 minutes
Size	260 kB
Frame width	720
Frame height	404
Frame rate	10 frames/second
Number of frames	55

**Table 3:** Sample spots in a video benchmark dataset.

Year	Name of the datasets	Number of videos	Number of classes/actions
2009	UCF11	1600	11
2009	UCF sports	150	10
2010	UCF50	50	10
2010	Olympic sports	800	16
2012	UCF101	13320	101
2014	Sports1-M	1133158	487
2018	Youtube8-M	6.1M	3862

**Table 4:** Experimental setup [24].

Hyper-parameters	Values
Hardware resource	Single CPU
Number of classes	10(Basketball, diving, golf, horse riding, kicking-front, running, skate boarding-front, swing-bench, walk-front, weight lifting)
Image/Frame size	227 × 227
Output size in fully connected layer	10 -no. of classes
Optimizer	SGDM - stochastic gradient descent with momentum
Epochs	6
Iterations per epoch	97
Maximum iterations	582
MiniBatch size	10
Initial learning rate	0.0001
Validation frequency	3
Kernel	3 × 3 filter
Stride	1
Activation unit	ReLU - rectified linear unit
Dropout	0.5

**Table 5:** Performance of various optimizers with the UCF101 and Sports1-M dataset.

Optimizers	Epochs	UCF101 dataset		Sports1-M dataset	
		Training accuracy (%)	Test accuracy (%)	Training accuracy (%)	Test accuracy (%)
SGDM	60	92.77	93.59	82.52	89.75
ADAM	50	87.38	83.59	83.90	80.71
NADAM	50	78.23	72.46	81.28	80.56
ADADelta	50	84.72	81.00	86.19	82.26
ADAGrad	50	87.04	84.62	84.39	81.17

**Table 6:** Comparison of various deep learning architectures with the proposed model for the UCF101 dataset.

Reference	Architectures	Activation function	Optimizer	Batch size	Epochs	Learning rate	Training accuracy (%)	Testing accuracy (%)
[26]	AlexNet	Softmax	SGDM	10	15	0.0001	90.87	92.68
[26]	GoogleNet	Softmax	SGDM	15	15	0.0001	90.29	91.67
[18]	VGG16	ReLu	SGDM	128	60	0.001	100	93.52
[27]	VGG19	ReLu	SGDM	128	60	0.001	100	93.33
[27]	CNN	Softmax	Adam	128	50	0.001	93.09	93.56
**Proposed model**	**Customized CNN**	**Softmax**	**SGDM**	**128**	**60**	**0.0001**	**92.77**	**93.59**

**Table 7:** Comparison of various deep learning architectures with the proposed model for the Sports1-M dataset.

Reference	Architectures	Activation function	Optimizer	Batch size	Epochs	Learning rate	Training accuracy (%)	Testing accuracy (%)
[26]	AlexNet	Softmax	SGDM	10	15	0.0001	87.14	89.25
[26]	GoogleNet	Softmax	SGDM	15	15	0.0001	88.04	90.58
[27]	VGG16	ReLu	SGDM	128	60	0.001	96.01	90.91
[27]	VGG19	ReLu	SGDM	128	60	0.001	97.05	91.74
[27]	CNN	Softmax	Adam	128	50	0.001	89.11	90.73
**Proposed model**	**Customized CNN**	**Softmax**	**SGDM**	**128**	**60**	**0.001**	**82.52**	**89.75**

**Table 8:** Hardware setup.

Peripheral/hardware	Model/version
Memory/RAM	Minimum 16 GB
Processor	Multicore Intel i9/i7/i5
Storage	At least 256 GB HDD preferably SSD

## Data Availability

The datasets are available in the UCF101 and Sport1-M repository.
